# Specific soil factors drive the differed stochastic assembly of rhizosphere and root endosphere fungal communities in pear trees across different habitats

**DOI:** 10.3389/fpls.2025.1549173

**Published:** 2025-04-01

**Authors:** Yunfeng Liu, Zhenzhou Wang, Xiang Sun, Xueli He, Yuxing Zhang

**Affiliations:** ^1^ College of Horticulture, Hebei Agricultural University, Baoding, China; ^2^ College of Life Sciences, Hebei University, Baoding, China

**Keywords:** fungal community, rhizosphere, root endosphere, assembly, ecological relationship

## Abstract

**Introduction:**

*Pyrus betulifolia* is tolerant to diverse environmental conditions and is commonly planted in infertile habitats (such as beaches and ridges) to conserve arable land for cereal crops. Symbiotic fungi in the rhizosphere and root endosphere benefit host plants by enhancing their resilience to nutritional deficiencies under stressful conditions. However, the mechanisms underlying the assembly of these symbiotic fungal communities in the roots of *P. betulifolia* across different habitats remain poorly understood.

**Methods:**

*Pyrus betulifolia* of 30-year-old were selected from five sites in northern China to investigate the assembly of fungal communities in the rhizosphere and root endosphere. Soil samples were collected to assess the heterogeneity of the environment surrounding each plant. Procrustes analysis, variance partitioning analysis, and ordination regression analysis were employed to explore the ecological relationships between soil factors and fungal community composition.

**Results:**

The rhizosphere fungal community exhibited higher richness, greater diversity and lower structural variability compared to the root endosphere. Additionally, the rhizosphere supported a fungal network with higher abundance and stronger connectivity than the root endosphere. The composition of fungal communities varies significantly among different regions. In both the rhizosphere and root endosphere fungal communities, the number of genera specific to mountainous regions was larger than those in plain areas and saline-alkali areas. Null model-based analyses indicated that the assembly of rhizosphere and root endosphere fungal communities in *P. betulifolia* was mainly governed by stochastic processes. Specifically, in non-saline-alkali soils, the assembly of rhizosphere fungi was primarily driven by dispersal limitation, whereas the assembly of root endosphere fungi was dominated by ecological drift. In saline-alkali soils, both rhizosphere and root endosphere fungal communities were primarily influenced by ecological drift.

**Conclusion:**

The assembly of root-associated fungal communities in *P. betulifolia* is not only driven by soil physicochemical properties but also influenced by root compartment niche and topography. Moreover, the impact intensity of the root compartment niche is greater than topography. Specifically, the assembly of the rhizosphere fungal community was primarily influenced by alkaline nitrogen (AN) and alkaline phosphatase (ALP), while the root endosphere fungal community was more strongly affected by pH and sucrase (SUC). These findings could provide valuable insights for the design of beneficial root-associated microbiomes to enhance fruit tree performance.

## 1 Introduction

The soil-root interface is a hotspot for microbial activity, where plant roots provide abundant organic compounds, attracting a diverse array of microorganisms. These microorganisms establish complex symbiotic relationships with plant roots. Along the soil-root continuum, plant roots organize soil microorganisms into three distinct microhabitats, based on differences in the plant-inhabiting niches: the rhizosphere (the microbial community surrounding the roots), the rhizoplane (the epiphytic microbial community on the root surface), and the root endosphere (the microbial community living inside the roots) (Lee et al., 2019). Among plant-associated microbial communities, fungi dominate and play a crucial role in plant health and functionality (Trivedi et al., 2020). In recent years, significant progress has been made in understanding the characteristics and assembly mechanisms of microbial communities in the rhizosphere and root endosphere. Different root niches support distinct microbial communities, and the process of community assembly is a key mechanism that determines microbial composition. This process is influenced either by the filtering effects of environmental parameters or by stochastic processes, such as random dispersal. While research on root-associated microorganisms has predominantly focused on grasses and crops (Latz et al., 2021), studies on woody plants, particularly pears, remain limited. Therefore, understanding the assembly rules of fungal communities in pears and their ecological relationships with soil physicochemical properties is crucial for advancing our knowledge of fruit trees ecosystem structure and function.

Rhizosphere fungi form symbiotic relationships with plants, directly influencing plant growth and health (Bakker et al., 2013). These fungi convert nutrients that are difficult for plant roots to absorb, such as organic phosphorus, into more accessible forms, thereby enhancing nutrient utilization efficiency in plants. The assembly of rhizosphere fungi is shaped by multiple factors, including soil environmental conditions and root compartment niches. On one hand, changes in the physicochemical properties of the soil can directly or indirectly alter the living conditions of rhizosphere fungi, affecting their assembly patterns. On the other hand, host plants interact with rhizosphere fungi through root exudates and other mechanisms, exerting either inducing or inhibitory effects on fungal assembly (Hassani et al., 2018). However, the primary factors influencing the assembly of rhizosphere fungi remain unclear.

The biological relationship between plants and root endophytes is symbiotic and co-evolutionary. These endophyte taxa are considered the “second genome” of plants and play a crucial role in the health of multicellular hosts (Trivedi et al., 2020). For example, mycorrhizal fungi expand the root system’s absorption area, enhancing water and phosphorus uptake (Basiru et al., 2023). Endophytic fungi such as *Paraphoma* sp. derived from the root system can help plants neutralize heavy metal toxicity and improve stress resistance. Investigating the composition and assembly process of fungal communities in the root endosphere of pear trees under different habitats, as well as their interrelationships with soil physicochemical properties, is crucial for comprehensively understanding the plant-microbe symbiotic system.

*Pyrus betulifolia* belongs to the deciduous trees of Rosaceae. As a wild species widely distributed in northern China, it is native to the deciduous forest areas in northern, central China and Tibet. Its root system is well-developed with numerous fibrous roots, and it has excellent abilities of drought tolerance, cold tolerance and salt-alkali stress tolerance (Li et al., 2016). Moreover, it can tolerate lime-induced iron chlorosis (Zhao et al., 2020). Due to its excellent grafting compatibility, *P. betulifolia* is the main rootstock in the commercial pear production in northern China, and also a crucial parental material in the cultivation of dwarf pear rootstocks and the resistance breeding work. Although *P. betulifolia* can adapt to various extreme soil types such as mountainous areas and saline -alkali lands, it is extremely sensitive to the changes in soil fertility. Therefore, regulating the soil physicochemical properties is of great significance for the growth and development of *P. betulifolia* plants and even the improvement of the pear yields above-ground. Traditional soil improvement methods, such as deep plowing and the application of organic fertilizers, are effective but costly. Adjusting the structure of the fungal community in the rhizosphere and endosphere, as a “green” strategy, can enhance the rhizosphere micro-ecological environment and optimize soil properties (Edwards et al., 2015). Previous studies have reported the soil physicochemical properties and fungal community composition of pear trees in different regions, but the research remains relatively superficial. For example, Huang et al. reported the effects of soil chemical properties and geographical distance on the composition of arbuscular mycorrhizal fungal communities in pear orchards (Huang et al., 2019). Currently, the role of the holobiome system composed of *P. betulifolia* and its microbial symbionts has received little attention. In this study, 30-year-old *P. betulifolia* pear trees were selected from five sites in Northern China to investigate the composition of rhizosphere and root endosphere fungal communities and their assembly processes. We formulated three hypotheses: (1) The root compartment niches (rhizosphere and root endosphere) of pear trees significantly influence the diversity and composition of fungal communities. (2) The assembly of rhizosphere and root endosphere fungal communities in pear trees is primarily governed by stochastic processes and is influenced by soil physicochemical factors. (3) Fungal community composition varied significantly across regions, with topography influencing fungal assembly. This study contributes to a better understanding of the relationship between plants and their microbiota, providing a foundation for engineering beneficial plant microbiota in sustainable agricultural production.

## 2 Materials and methods

### 2.1 Experimental sites

Pear’s root and rhizosphere soil samples were collected in May 2023 from five pear-growing regions in Hebei Province, China: WX, XJ, BT, QY, and YT. At the regional scale, topography is a crucial determinant of the patterns of species diversity and species composition within forest communities. Moreover, various habitat types have often been proven to be defined by topographic features, such as altitude (Wang et al., 2022). According to the geomorphic features and altitudes, five regions were classified into two types: the mountainous type, which includes YT and QY regions, and the plain type, which encompasses BT region, WX region, and XJ region. Since *P. betulaefolia* plants in YT region mostly grow in valley areas, while a large number of pear trees in QY region are planted at relatively high altitudes, the altitudes of the sampling sites in YT region are relatively low, and those in QY region are relatively high. As the samples in this study were collected from the salinized soil area of BT region, and according to the measurement results of soil pH, which were significantly higher than those of other regions, BT region was classified as a saline-alkali land type. Finally, these three different types of ecological environments were uniformly defined as habitat types, namely the mountainous type, the plain type, and the saline-alkali land type. These regions share a temperate semi-arid monsoon climate with distinct seasonal variations. Detailed regional information was provided in **Supplementary Table S1**.

### 2.2 Collection strategy and laboratory process of samples

Three plots of 30 m × 20 m were established at each site (WX, XJ, QY, and BT), and two plots were set up at YT, for a total of 14 plots, in May, 2023. Nine healthy 30-year-old pear trees at the full fruiting stage were randomly selected in each plot (126 individuals in total). Dead branches and leaves around each tree were removed. Root and rhizosphere soil samples were collected from each selected tree using a shovel that was sprayed with 75% ethanol and wiped with sterilized paper before use. Fine roots (approximately 50 g) were carefully picked from a hole 30 cm deep, 30–50 cm away from the main trunk with sterile plastic bags. Rhizosphere soil (approximately 1000 g) was gently shaken off the roots and collected with sterile plastic bags. The root and soil samples were labeled and transported to the laboratory on ice immediately. Rhizosphere soil for DNA extraction was gently brushed off the roots with a fine brush, collected into 5 mL centrifuge tubes, and stored at -80°C. Root samples were brushed and washed with deionized water, then surface-sterilized with 70% ethanol for 5 minutes and 5% sodium hypochlorite for 2 minutes, followed by rinsing with sterile distilled water. These samples were then frozen in liquid nitrogen and stored at -80°C for DNA extraction. Fifty grams of bagged rhizosphere soil were stored at 4°C for subsequent enzyme activity assays. The remaining soil was air-dried, sieved through a 2-mm mesh, and used for physical and chemical property measurements.

### 2.3 Extraction of total DNA and high-throughput sequencing

Root and rhizosphere soil samples of 0.1 g each were used for DNA extraction. Total genomic DNA was extracted using the E.Z.N.A.^®^Soil DNA Kit (Omega Bio-tek) following the manufacturer’s instructions. DNA concentration and purity were assessed using a NanoDrop 2000 (Thermo Fisher Scientific, USA) and checked for quality by 1% agarose gel electrophoresis. PCR amplification of the fungal ITS1 region was performed using barcoded primers ITS1F (5’-CTTGGTCATTTAGAGGAAGTAA-3’) and ITS2R (5’-GCTGCGTTCTTCATCGATGC-3’). The PCR mixture contained 4 μL of 5×TransStart FastPfu buffer, 2 μL of 2.5 mM dNTPs, 0.8 μL of each primer (5 μM), 0.4 μL of TransStart FastPfu DNA polymerase, 10 ng of DNA, and nuclease-free water to a final volume of 20 μL. Amplification conditions were as follows: 95°C for 3 min, followed by 27 cycles of 95°C for 30 s, 55°C for 30 s, and 72°C for 30 s, with a final extension at 72°C for 10 min. PCR products were purified by 2% agarose gel electrophoresis and extracted using the AxyPrep DNA Gel Extraction Kit (Axygen Biosciences, Union City, CA, USA). The purified products were quantified using the Quantus™ Fluorometer (Promega, USA). Library construction was performed using the NEXTFLEX Rapid DNA-Seq Kit, including adapter ligation, magnetic bead selection, PCR enrichment, and bead recovery. Sequencing was conducted on the Illumina PE300 platform (Shanghai Meiji Biomedical Technology Co., Ltd.), and raw data were uploaded to the NCBI SRA database (PRJNA1161009, PRJNA1160469).

Quality control of paired-end raw sequencing reads was conducted using fastp (Chen et al., 2018) (https://github.com/OpenGene/fastp, version 0.19.6). Read merging was performed with FLASH (http://www.cbcb.umd.edu/software/flash, version 1.2.11), filtering out bases with a quality score below 20 at read tails using a 50 bp sliding window. Reads shorter than 50 bp or containing N bases were discarded. Paired reads were merged with a minimum overlap of 10 bp and a maximum allowed mismatch ratio of 0.2. Sequences were filtered based on barcode (0 mismatches) and primer mismatches (up to 2 mismatches). Quality-controlled and merged sequences were clustered into Operational Taxonomic Units (OTUs) with 97% similarity using UPARSE v7.1 (Edgar, 2013) (http://drive5.com/uparse/), and chimeric sequences were removed. Sequences annotated as non-fungi organisms were excluded. Samples were rarefied to the minimum sequence count. OTU taxonomic classification was conducted using the RDP classifier (http://rdp.cme.msu.edu/, version 2.11) against UNITE database (version 8.0). Functional guild annotation was performed using FUNGuild software v1.0 (http://www.funguild.org/).

### 2.4 Soil physicochemical properties analysis

SM was measured using a FieldScout TDR 350 (Spectrum, Aurora, Illinois, US) soil moisture meter. Soil pH was determined with a portable pH meter (pH 3000, STEP Systems GmbH, Germany). OC was assessed by loss-on-ignition in a TMF-4-10 T muffle furnace (Gemtop, Shanghai, China) at 550°C for 4 h (Heiri et al., 2001). TN was measured using the semi-micro Kjeldahl method, and TP was quantified by sulfuric acid-hypochlorite digestion (Valderrama, 1981). Alkaline nitrogen (AN) and AP were measured using the alkaline diffusion method and sodium bicarbonate extraction-molybdenum antimony colorimetric method, respectively (Bever et al., 1996; Tarafdar and Marschner, 1994). AK was determined by NH_4_OAc extraction-flame photometry. EC was measured with a DDS-307W conductivity meter (Lida Instruments, Shanghai, China). The C/N ratio was calculated from soil OC and TN. ALP activity was measured using the modified Bremner and Tabatabai method (Tarafdar and Marschner, 1994), assessing the conversion of pNPP (µg/g) per gram of soil per hour. UR activity was assessed by the method described by Kandeler and Gerber (1988), measuring NH_3_-N production (µg) from urea decomposition per gram of soil per hour. SUC activity was assessed using the 3,5-dinitrosalicylic acid method, measuring glucose (mg/g) produced from sucrose hydrolysis per gram of soil. Altitude and latitude-longitude data were obtained from a GPS device. MAT and MAP were sourced from the China Meteorological Data Network (http://cdc.cma.gov.cn).

### 2.5 Statistical analysis

Alpha diversity was calculated using mothur (Rzehak et al., 2024) software (http://www.mothur.org/wiki/Calculators), with differences analyzed using the Wilcoxon rank-sum test (*P* < 0.05). PCoA based on Bray-Curtis distance was used to assess the similarity in fungal community structure, while Beta diversity analysis evaluated within-group dispersion of rhizosphere and root endosphere fungal communities. PERMANOVA non-parametric tests and ANOSIM similarity analysis were conducted to explore differences between rhizosphere and root endosphere fungi, as well as variations across different regions. Based on the results of the taxonomic analysis, the species compositions at the phylum and class levels of different groups were analyzed. The Wilcoxon rank-sum test was used to analyze whether microbial community relative abundances were significantly different between rhizosphere and root endosphere (*P* < 0.05). Differences among regions were analyzed using a Kruskal-Wallis H test (*P* < 0.05). R (version 3.3.1) software was used to achieve the Venn diagram at the genus level. Circos software (Circos-0.67-7, http://circos.ca/) was used to visualize the relationships between rhizocompartment types or regions types and the relative abundances of fungal trophic modes. Soil physicochemical properties were analyzed using SPSS 24.0, and mean values were compared using Duncan’s test (*P* < 0.05). Procrustes analysis via PCA plots was used to assess correlations between fungal communities and soil properties. VPA investigated the effects of soil, geographical, and climatic factors on fungal communities. Mantel analysis in R 3.3.1 with the vegan package (version 2.4.3) examined the influence of soil physicochemical properties on microbial structure. Spearman correlation was used to assess the association between soil physicochemical properties and fungal phyla.

Network parameters were analyzed using R packages (igraph, psych, Hmisc, vegan, dplyr, reshape2), and network modularity was assessed in Gephi. Microbial diversity and ecological evolution were studied within a deterministic versus stochastic framework. The βNTI index (based on βMNTD and phylogenetic quantification) was used to determine process dominance: |βNTI| ≥ 2 indicated deterministic processes, while |βNTI| < 2 indicated stochastic processes. The relative contributions of deterministic (homogeneous and heterogeneous selection) and stochastic processes (dispersal limitation, homogeneous dispersal, ecological drift) were quantified using βNTI and RCBray.

## 3 Results

### 3.1 Analysis of rhizosphere soil physicochemical properties

**Figure 1:**
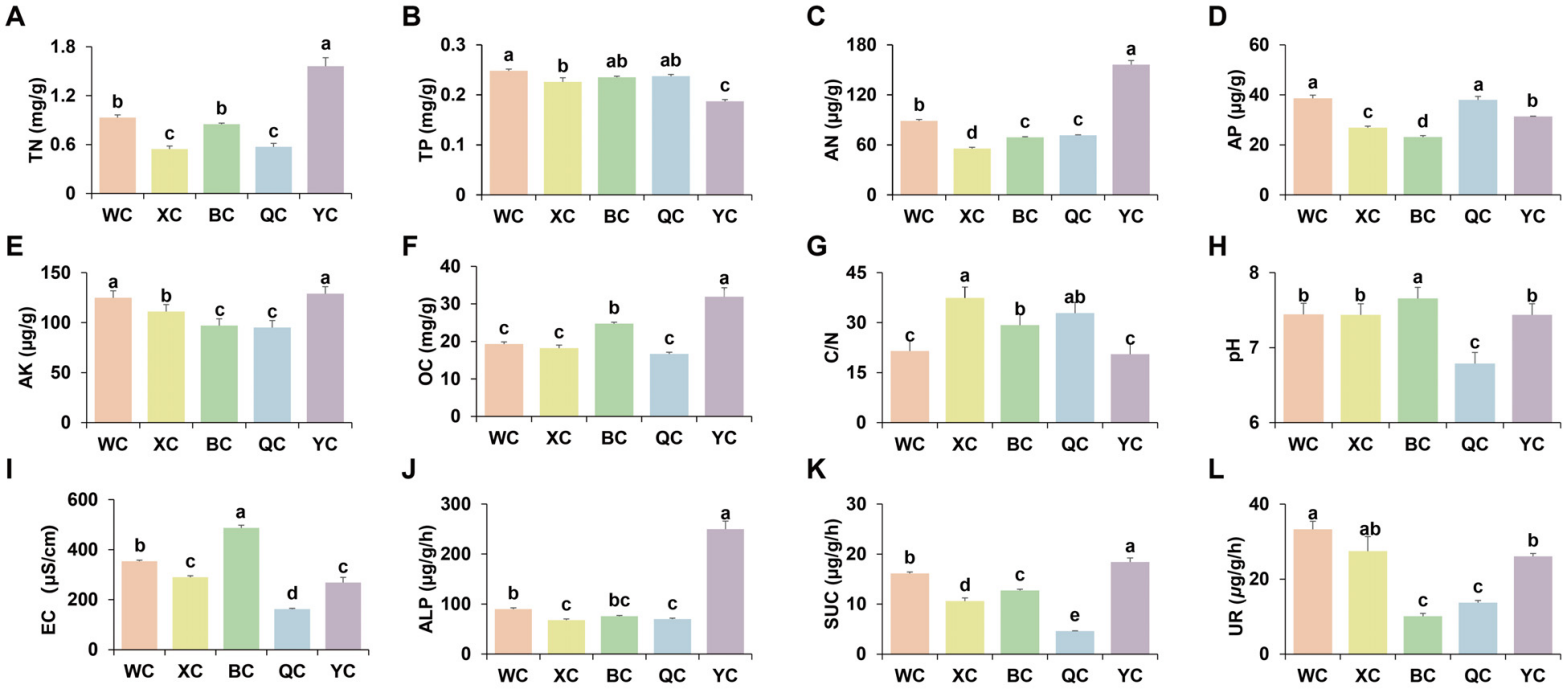
Characteristics of physicochemical properties and enzyme activity of the pear trees rhizospheric soil among different regions. The error bars illustrate the standard error of the mean. Different lowercase letter above the error bars indicates significant difference at *P* < 0.05. **(A)** TN. **(B)** TP. **(C)** AN. **(D)** AP. **(E)** AK. **(F)** OC. **(G)** C/N: OC/TN. **(H)** pH. **(I)** EC. **(J)** ALP. **(K)** SUC. **(L)** UR. The WC, XC, BC, QC and YC represent rhizosphere soils from WX, XJ, BT, QY and YT regions, respectively.

Analysis of the physicochemical properties of rhizosphere soil across regions revealed that YT had the highest levels of soil TN (1.56 mg/g), AN (156.14 μg/g), and OC (31.92 mg/g), but the lowest TP (0.19 mg/g) (**Figures 1A–C, F**). The C/N ratio was highest in XJ (37.42), significantly exceeding that of WX, BT, and YT, with no significant difference from QY (**Figure 1G**). WX had the highest TP and AP at 0.25 mg/g and 38.59 μg/g, respectively (**Figure 1B, D**). Soil AK was significantly higher in WX and YT, at 124.83 μg/g and 129 μg/g, respectively, compared to other regions (**Figure 1E**). Soil pH was the highest in BT at 8.22 and lowest in QY at 6.79 (**Figure 1H**). Soil EC was the highest in BT (486.67 μS/cm) and lowest in QY (162.25 μS/cm) (**Figure 1I**). Soil ALP activity was the highest in YT at 249.87 μg/g/h (**Figure 1J**). Soil UR activity was the highest in WX (33.28 μg/g/h) and XJ (27.44 μg/g/h), followed by YT (26.09 μg/g/h), with BT and QY having the lowest values at 10.11 μg/g/h and 13.74 μg/g/h, respectively (**Figure 1L**). SUC activity was the highest in YT at 18.41 μg/g/h and lowest in QY at 4.61 μg/g/h (**Figure 1K**). SM was highest in BT (144.29%vwc) and lowest in QY (77.98%vwc) (**Supplementary Figure S1**).

### 3.2 Diversity and symbiotic patterns of rhizosphere and root endosphere fungi

**Figure 2:**
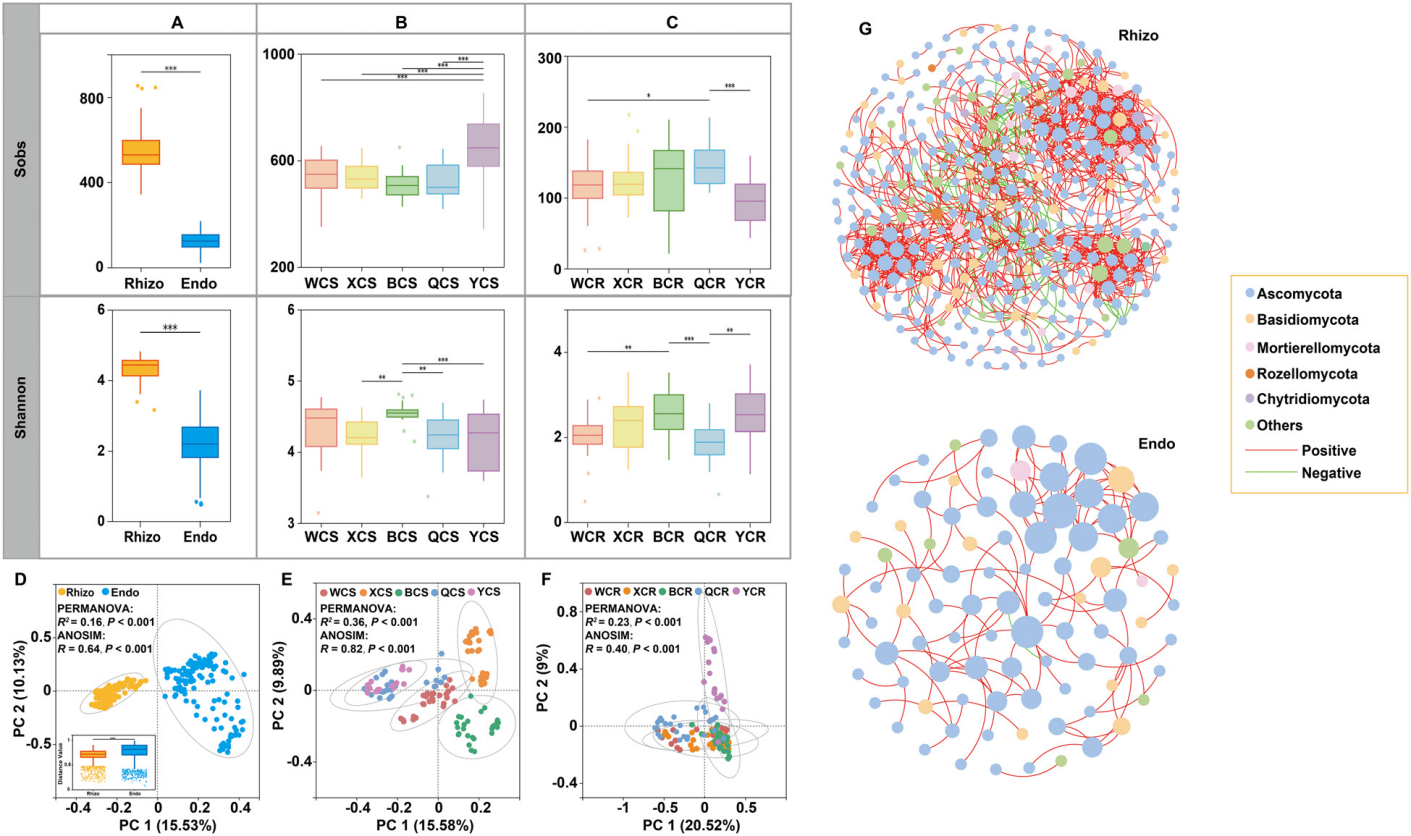
The α diversity and β diversity of fungal communities inhabiting the rhizosphere (Rhizo) and root endosphere (Endo). **(A)** Sobs and Shannon indices of fungal communities in the rhizosphere and root endosphere. **(B)** Sobs and Shannon indices of fungal communities in the rhizosphere of different regions. **(C)** Sobs and Shannon indices of fungal communities in the root endosphere of different regions. **(D)** PCoA plot depicting the β diversity patterns of rhizospheric and root endospheric fungal communities. **(E)** PCoA plot depicting the β diversity patterns of rhizospheric fungal communities of different regions. **(F)** PCoA plot depicting the β diversity patterns of root endospheric fungal communities of different regions. **(G)** Co-occurrence networks of fungal communities in the rhizosphere (Rhizo) and root endosphere (Endo). Nodes represent fungal OTUs. Edges represent significant interactive correlations between pairs of OTUs. Node colors represent fungal OTUs species information (phylum level) and the size of nodes corresponds to the relative abundances of specific fungus. Red edges indicate positive relationships, and green edges indicate negative relationships. *P < 0.05; **P < 0.01; ***P < 0.001. WCS (WCR), XCS (XCR), BCS (BCR), QCS (QCR) and YCS (YCR) represent rhizosphere (root endosphere) fungi in WX, XJ, BT, QY, and YT regions, respectively.

Alpha and Beta diversity metrics were used to assess fungal community heterogeneity. The abundance and Shannon index (α-diversity) of rhizosphere fungi were significantly higher than those of root endosphere fungi (*P* < 0.001). Specifically, the Sobs index for rhizosphere fungi in YT was significantly higher than in other regions (*P* < 0.001). The Shannon index of rhizosphere fungi in BT was significantly higher than in XJ, QY and YT (*P* < 0.001). For root endosphere fungi, the Sobs index in QY was the highest, significantly greater than in WX (*P* < 0.05) and YT (*P* < 0.001). The Shannon index of root endosphere fungi was significantly higher in BT than in WX (*P* < 0.01) and QY (*P* < 0.001). Additionally, the Shannon index of root endosphere fungi was significantly higher in YT than in QY (*P* < 0.01) (**Figures 2A–C**).

PCoA based on Bray-Curtis distance revealed that rhizosphere and root endosphere fungal communities formed two distinct clusters along the first coordinate axis, which explained 25.66% of the total variation, indicating distinct spatial differentiation (Permutational multivariate analysis of variance (PERMANOVA): *R²* = 0.16, *P* < 0.001; ANOSIM: *R* = 0.64, *P* < 0.001). Root endosphere fungal communities exhibited significantly higher variation in sample classification compared to rhizosphere fungi (*P* < 0.001). PCoA analysis of rhizosphere fungi across regions showed significant compositional differences (PERMANOVA: *R²* = 0.36, *P* < 0.001; ANOSIM: *R* = 0.82, *P* < 0.001). Rhizosphere fungal communities in QY, YT, and WX did not significantly separate along the first coordinate axis but were clearly distinct from those in BT and XJ. Rhizosphere fungi in BT and XJ formed independent clusters along the second coordinate axis, indicating differences from other regions. For root endosphere fungi, PCoA analysis also revealed significant compositional differences among regions (PERMANOVA: *R²* = 0.23, *P* < 0.001; ANOSIM: *R* = 0.40, *P* < 0.001) (**Figures 2D–F**). PERMANOVA of fungal data showed that variations in microbial communities were influenced by both region types and root compartment niches (*R^2^* = 0.40, *P* < 0.001). In addition, niches (*R^2^* = 0.16, *P* < 0.001) explained more differences in the microbial community than region type (*R^2^* = 0.14, *P* < 0.001). Particularly, region types (*R^2^* = 0.14, *P* < 0.001) explained more differences in the microbial community than topography (*R^2^* = 0.08, *P* < 0.001). All regions rendered rhizosphere and root endosphere fungal microbiota significantly dissimilar from each other (*P* values listed in **Table 1**).

**Table 1:** R^2^ and *P* values calculated by PERMANOVA for the variance of fungal communities among different regions.

Factors	Results by factor		Results by regions/niche		
	R^2^	*P* value^a^		R^2^	*P* value^a^
Root compartment niche (Rhizo *vs* Endo)	0.16	0.001	WX	0.28	0.001
			XJ	0.25	0.001
			BT	0.29	0.001
			QY	0.30	0.001
			YT	0.35	0.001
region type	0.14	0.001	Rhizo	0.36	0.001
			Endo	0.23	0.001
Topography	0.08	0.001	Rhizo	0.20	0.001
			Endo	0.11	0.001
Compartment niche×region type	0.40	0.001			
Compartment niche×topography	0.28	0.001			

Rhizo, rhizosphere; Endo, root endosphere.

Co-occurrence networks analysis revealed that the rhizosphere fungal community formed a highly interconnected network with a larger scale (nodes = 351) and higher connectivity (edges = 1171) (**Supplementary Table S2**). Positive correlations dominated in both networks, accounting for 87.36% and 99.19%, respectively, while negative correlations were less common (12.64% and 0.81%) (**Figure 2G**). Comparative analysis across regions showed notable differences in network structures, despite high connectivity of rhizosphere and root endosphere fungi in various regions (**Supplementary Figure S2**). In BT, the rhizosphere fungal network had the most nodes (nodes = 279), whereas XJ had the highest number of edges, clustering coefficient, and network density (edges = 4434; clustering coefficient = 0.575; network density = 0.146), but the lowest modularity (modularity = 0.228). Conversely, YT’s rhizosphere fungal network had the fewest nodes but the highest modularity (nodes = 175; modularity = 0.424). WX’s rhizosphere fungal network showed lower connectivity and clustering coefficient (edges = 1401; clustering coefficient = 0.399). For root endosphere fungi, YT’s network had the most nodes (nodes = 97), while XJ led in edges, clustering coefficient, and network density (edges = 548; clustering coefficient = 0.663; network density = 0.173). WX’s root endosphere fungal network had the lowest node connectivity but the highest modularity (nodes = 38; edges = 38; modularity = 0.798) (**Supplementary Table S2**).

### 3.3 Taxonomic and functional composition of rhizosphere and root endosphere fungi

**Figure 3:**
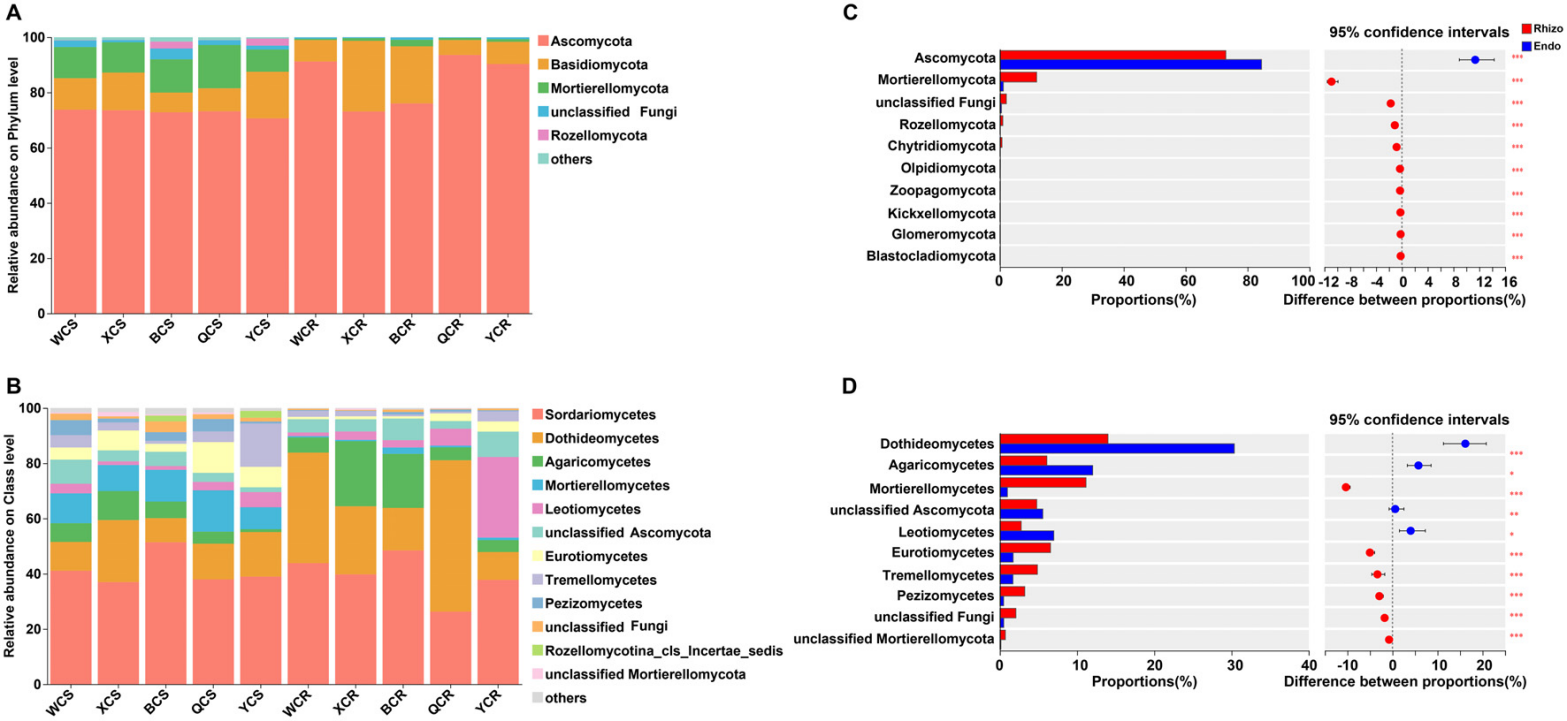
Fungal composition of the rhizosphere (S) and root endosphere (R) of *P. betulifolia*: relative abundances of fungal community structure at the phyla **(A)** and class **(B)** levels. **(C)** and **(D)** show taxa differences of fungi between the rhizosphere and root endosphere at the phyla and class levels, respectively. The vertical axis shows taxa at class level with mean sums in the top 10, and different colored boxes indicate different groups. The horizontal axis represents the average relative abundance of taxa. *P < 0.05; **P < 0.01; ***P < 0.001, Rhizo and Endo, Rhizosphere and root endosphere, respectively.

Taxonomic analysis revealed the OTUs were classified into 14 phyla, 46 classes, 111 orders, 249 families, and 560 genera. The dominant fungal phyla were *Ascomycota* (70.63–93.58%), *Basidiomycota* (5.43–25.61%) and *Mortierellomycota* (8.04–15.66%) (**Figure 3A**). At the class level, the dominant classes were *Sordariomycetes* (36.98–51.42%) and *Dothideomycetes* (8.72–22.46%). Notably, *Dothideomycetes* (54.82%) was the most abundant in the root endosphere of QY (**Figure 3B**). Fungal taxonomic distributions showed significant differences between the rhizosphere and root endosphere microhabitats. *Ascomycota* was significantly (*P* < 0.05) enriched in the root endosphere, while *Mortierellomycetes* was depleted in this compartment compared to the rhizosphere (**Figure 3C**). *Dothideomycetes*, *Agaricomycetes*, and *Leotiomycetes* were significantly (*P* < 0.05) enriched in the root endosphere, whereas *Mortierellomycetes*, *Eurotiomycetes*, *Tremellomycetes* and *Pezizomycetes* were depleted, as compared to the rhizosphere (**Figure 3D**). Region-specific patterns were observed, with *Leotiomycetes* (29.17%) being significantly (*P* < 0.05) enriched in the root endosphere of Yutian (YT), and *Tremellomycetes* (15.70%) enriched in the rhizosphere of YT. *Agaricomycetes* was significantly (*P* < 0.05) enriched in the root endosphere of XJ and BT (**Supplementary Figure S3**).

Venn analysis revealed that the rhizosphere and root endosphere fungal genera numbered 411 and 544, respectively, with 395 shared genera, representing 53.55% of the total. The rhizosphere contained 149 unique genera, while the root endosphere had only 16 unique genera, indicating substantial differences in community composition. Regional comparisons revealed that 298 genera were shared across regions in the rhizosphere (30.13% of total), while the number of unique genera varied across regions: YT and QY had 21 and 7 unique genera (5.64% and 3.80%, respectively); BT had 6 unique genera (2.63%); WX and XJ had 3 and 5 unique genera (1.57% and 1.88%, respectively). For root endosphere fungi, 101 unique genera were shared across regions (10.14% of total), with YT and QY having the most unique genera (29 and 23, respectively), followed by WX (21), XJ (18), and BT (15) (**Supplementary Figure S4**).

Functional annotation of fungal taxa using the FunGuild database categorized fungi into three primary trophic modes: pathotrophs, symbiotrophs, and saprotrophs. The major trophic modes were further divided into specific guilds, including arbuscular mycorrhizal fungi, plant pathogens, and animal pathogens (Schmidt et al., 2019). In terms of trophic modes, saprotrophs (38.49%) and pathotroph-saprotroph-symbiotrophs (29.4%) dominated both rhizosphere and root endosphere fungal assemblages (**Supplementary Figure S5A**). No significant differences in trophic modes were observed among rhizosphere fungi across regions (**Supplementary Figure S5B**). Notably, pathotroph-saprotrophs accounted for a larger proportion (32%) in the composition of root endophytic fungi in YT compared to other regions (**Supplementary Figure S5C**). From the perspective of fungal guilds, undefined saprotrophs (22.00%–29.97%) and unknown fungi (10.52%–32.98%) were predominant in both the rhizosphere and root endosphere across the five regions. Unknown fungi (32.98%) were the most abundant in the root endosphere of BT, while wood saprotrophs (37.92%) were most abundant in the root endosphere of QY. The Endophyte-Plant Pathogen-Undefined Saprotroph guild (26.92%) was a prominent category in the root endosphere of YT. Ectomycorrhizal fungi and endophytes were found in both rhizosphere and root endosphere across the five regions but in small quantities. Ericoid Mycorrhizal fungi were present in the rhizosphere and root endosphere of YT, WX, and QY, particularly in the QY root endosphere **(Supplementary Figures S5D, E).**

### 3.4 Ecological evolution of fungal community assemblage aggregation in the rhizosphere and root endosphere

**Figure 4:**
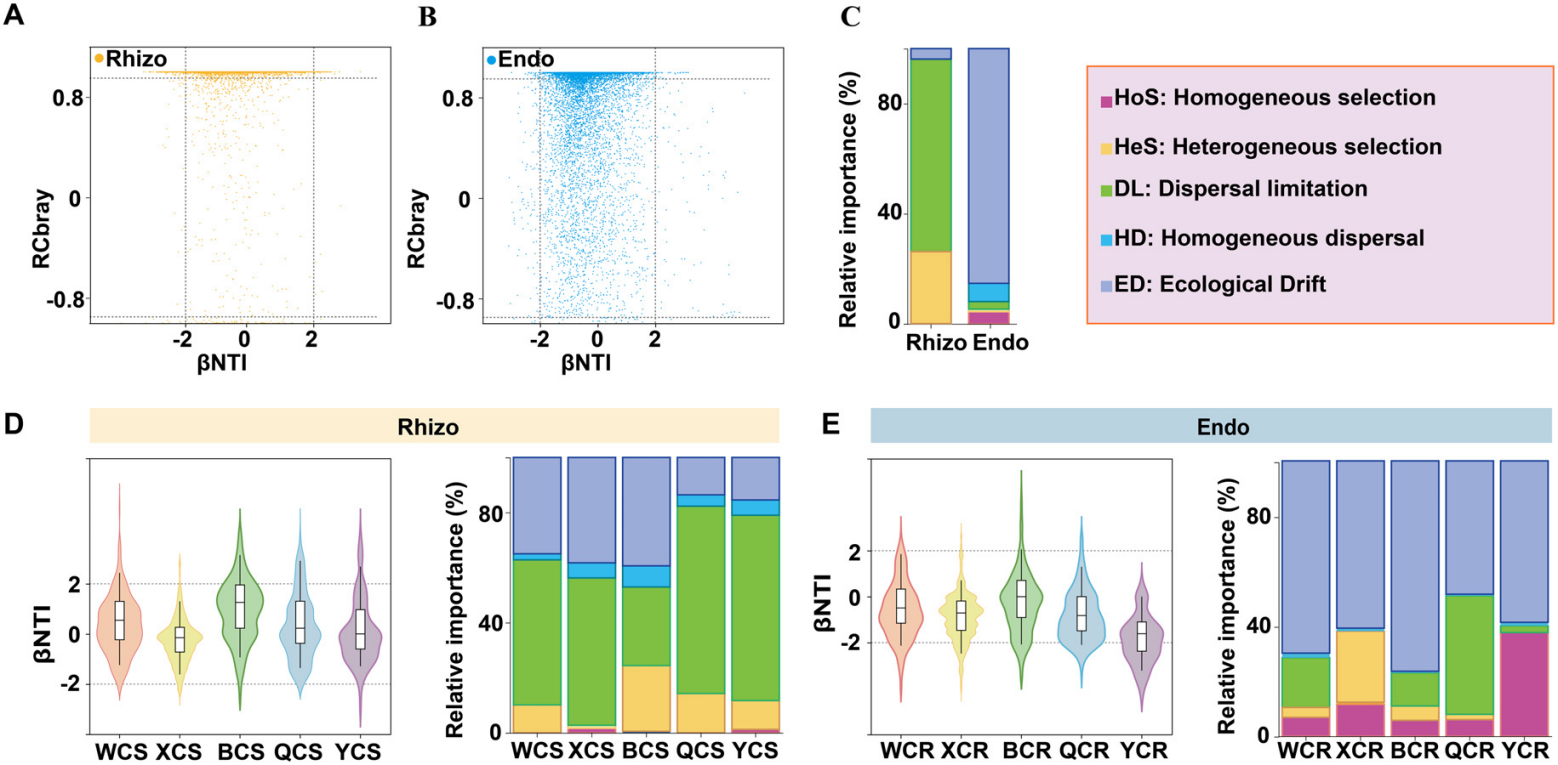
The relative importance of ecological processes that determine fungal community assembly in the rhizosphere (Rhizo) and root endosphere (Endo). **(A)** Variation in the βNTI and RCBray for fungal communities in the rhizosphere. **(B)** Variation in the βNTI and RCBray for fungal communities in the root endosphere. **(C)** The percentages depicted in stacked bar charts represent the proportions at which homogeneous selection, heterogeneous selection, dispersal limitation, homogenizing dispersal and ecological drift contribute to fungal community assembly. **(D)** Variation in the βNTI and RCBray for fungal communities in the rhizosphere of different regions. **(E)** Variation in the βNTI and RCBray for fungal communities in the root endosphere of different regions. βNTI > 2 indicates variable selection, and βNTI < –2 indicates homogenous selection. |βNTI| < 2 and RCBray < –0.95 indicate homogenous dispersal. |βNTI| < 2 and RCBray > 0.95 indicate dispersal limitation. |βNTI| < 2 and |RCBray| < 0.95 mainly indicate ecological drift.

The ecological and evolutionary mechanisms driving the assembly of rhizosphere and root endosphere fungal communities were explored using the βNTI and Raup-Crick (RC-Bray) models based on Bray-Curtis dissimilarity. The results revealed that stochastic processes predominated in both community types. Specifically, rhizosphere fungi were mainly influenced by dispersal limitation (72.68%), while root endosphere fungi were primarily shaped by ecological drift (84.24%) (**Figures 4A–C**). Regional analysis showed that the assembly of rhizosphere fungi in WX, XJ, QY, and YT was primarily controlled by dispersal limitation, contributing 52.67%, 53.50%, 68.04%, and 67.28%, respectively, to microbial community structure. In contrast, the assembly of rhizosphere fungi in BT was mainly driven by ecological drift (39.37%) (**Figure 4D**). For root endosphere fungi, ecological drift dominated across all regions, contributing 69.82%, 60.77%, 76.41%, 48.42%, and 58.64% in WX, XJ, BT, QY, and YT, respectively. Notably, in the QY region, dispersal limitation played a significant role, contributing 43.07%, which was nearly equivalent to the effect of ecological drift. Compared with other regions, homogeneous selection occupied a larger proportion (about 37.65%) of the root endosphere fungal assemblage in YT (**Figure 4E**).

### 3.5 Relationship between fungal assemblages in rhizosphere and root endosphere, and rhizosphere soil physicochemical properties

**Figure 5:**
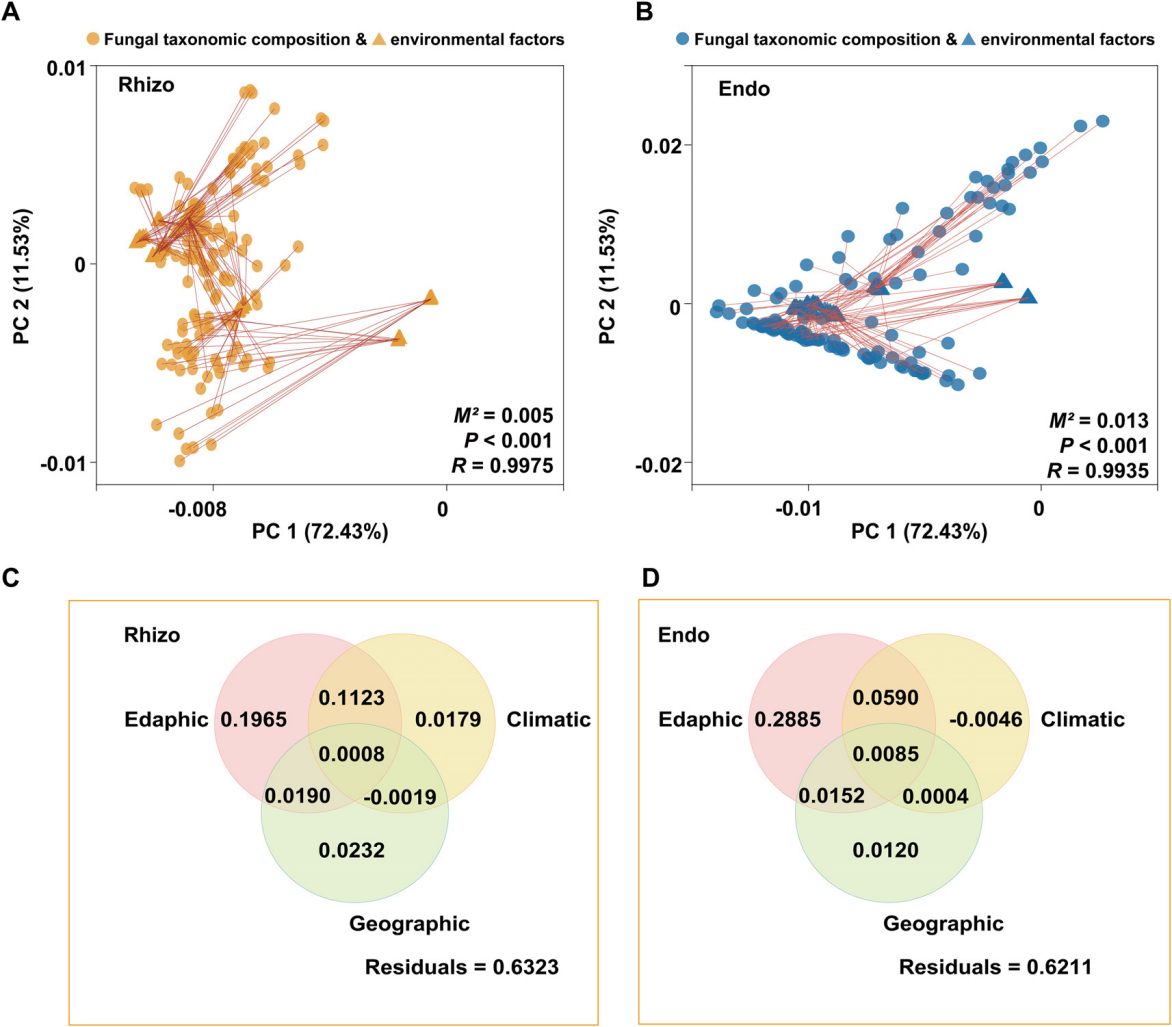
Analysis of correlation between the rhizospheric (Rhizo) and root endospheric (Endo) fungal taxonomic compositions and environmental factors. **(A)** Procrustes analysis of the rhizosphere fungal taxonomic compositions and environmental factors. **(B)** Procrustes analysis of the root endosphere fungal taxonomic compositions and environmental factors. *M^2^* indicates the sum of the squared distances between matched sample pairs. *R* represents the correlation in a symmetric Procrustes rotation; the *P* value is determined from 999 labeled permutations. **(C)** Variance partitioning analysis (VPA) of the relative contributions of edaphic (TN, TP, AN, AP, AK, OC, C/N, pH, EC, ALP, SUC, UR, SM), geographic (Altitude), and climatic (MAT and MAP) variables to the rhizosphere fungal taxonomic compositions. **(D)** Variance partitioning analysis (VPA) of the relative contributions of edaphic (TN, TP, AN, AP, AK, OC, C/N, pH, EC, ALP, SUC, UR, SM), geographic (Altitude), and climatic (MAT and MAP) variables to the root endosphere fungal taxonomic compositions.

**Figure 6:**
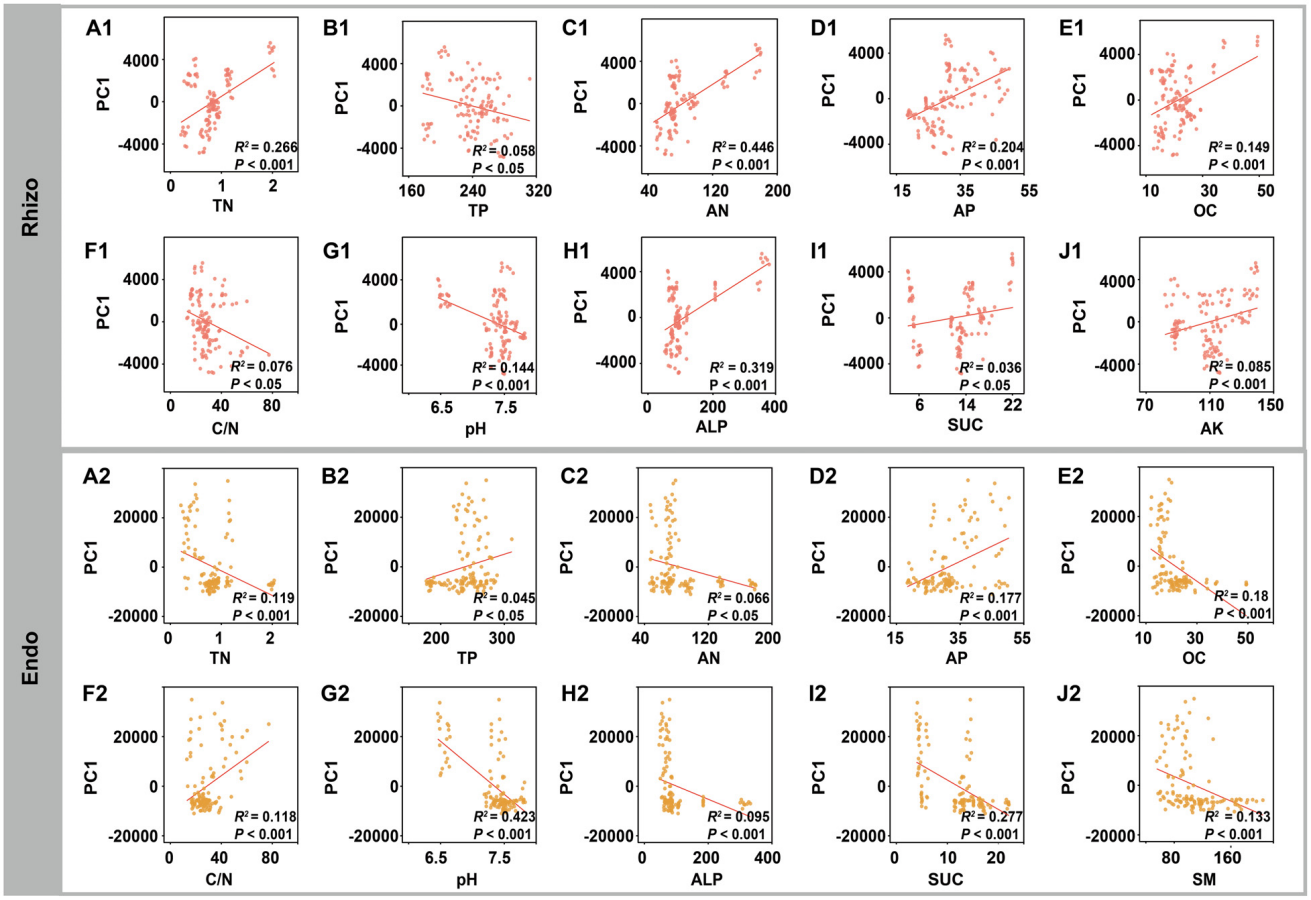
Relationships between the rhizosphere (Rhizo) and root endosphere (Endo) fungal taxonomic compositions and specific soil physicochemical properties. Linear regression model fittings illustrated significant relationships between the rhizosphere fungal taxonomic compositions and **(A1)** TN, **(B1)** TP, **(C1)** AN, **(D1)** AP, **(E1)** OC, **(F1)** C/N ratio, **(G1)** pH, **(H1)** ALP, **(I1)** SUC, and **(J1)** AK. Linear regression model fittings illustrated significant relationships between the root endosphere fungal taxonomic compositions and **(A2)** TN, **(B2)** TP, **(C2)** AN, **(D2)** AP, **(E2)** OC, **(F2)** C/N ratio, **(G2)** pH, **(H2)** ALP, **(I2)** SUC, and **(J2)** SM. *R* ^2^ represents the deviance explained by the linear regression model.

Procrustes analysis was used to compare the alignment between rhizosphere and root endosphere fungal community structures and environmental factors (e.g. soil properties, geography, and climate), revealing significant correlations with environmental factors (*P* < 0.001) (**Figures 5A, B**). Variance partitioning analysis (VPA) further indicated that rhizosphere soil physicochemical properties were key determinants of fungal community composition in both rhizosphere and root endosphere, explaining 32.86% and 37.12% of the variance, respectively (**Figures 5C, D**). Mantel analysis demonstrated significant relationships between fungal communities and rhizosphere soil physicochemical properties (**Supplementary Figure S6**). To reveal potential relationships, ordination regression analysis showed significant correlations (*P* < 0.05) between rhizosphere fungal composition and rhizosphere soil factors (TN, TP, AN, AP, OC, C/N, pH, ALP, SUC, and AK), except UR and SM. Similarly, root endosphere fungi displayed significant correlations (*P* < 0.05) with TN, TP, AN, AP, OC, C/N, pH, ALP, SUC, and SM, excluding UR and AK. In specific, the assembly of rhizosphere fungi community was mainly affected by AN (44.6%) and ALP (31.9%), while the assembly of root endosphere fungi was primarily affected by pH (42.3%) and SUC (27.7%) (**Figure 6**).

Spearman correlation analysis revealed that rhizosphere soil physicochemical properties had a stronger association with the relative abundances of major fungal orders in the rhizosphere community compared to the root endosphere. Correlation heatmap showed that pH was significantly positively correlated with the relative abundances of *Tubeufiales*, *Glomerellales*, and *Capnodiales*, but was negatively related to the relative abundances of *Pleosporales*, *Sebacinales*, *Cystofilobasidiales*, and *Chaetothyriales* in root endosphere of pear trees. In addition, pH was also significantly negatively correlated with the relative abundances of *Filobasidiales*, *Eurotiales*, and Microascales, but was positively related to the relative abundances of *Hypocreales* in rhizosphere of pear trees. These results indicated that the above-mentioned fungal taxa prefer the pH of the habitat. Furthermore, in terms of root endosphere of pear trees, the relative abundances of *Glomerellales*, *Tubeufiales*, and *Capnodiales* had a negative relationship with the contents of AP and UR, but had a positive relationship with the content of OC, pH and SM. Interestingly, *Pleosporales* showed an opposite correlation pattern with the above-mentioned taxa. In terms of rhizosphere of pear trees, the relative abundances of *Cystofilobasidiales*, and *Filobasidiales* had a positive relationship with the contents of AK, UR, SUC, ALP, TN, AN, and AP, but had a negative relationship with the content of pH and C/N (**Supplementary Figure S7**).

## 4 Discussion

### 4.1 Diversity and community structure of fungal communities in the rhizosphere and root endosphere

Fungal communities in the rhizosphere and root endosphere form a diverse micro-ecosystem surrounding and inhabiting plant roots, playing a critical role in assessing rhizosphere soil health and crop growth (Tan et al., 2017). A significant decline in the Sobs and Shannon indices of fungal communities from the rhizosphere to the root endosphere indicated that the barrier between these two compartments in pear trees exerts a strong filtering effect on the recruitment of specific microorganisms (**Figure 2A**) (Lumibao et al., 2020). This finding aligned with the theory of plant-microbiota coevolution, which posited that plants attract and select beneficial microbiota through the release of signaling molecules, immune system activation, and the provision of specialized nutrients and habitat types, thereby exerting selective pressure. Microbiota capable of recognizing signaling molecules and colonizing specific niches are preferentially enriched, while others are filtered out (Boyno and Demir, 2022). The structural variability of fungal communities in the root endosphere was greater than that in the rhizosphere, suggesting that the processes of colonization and community formation in the root endosphere are more variable (**Figure 2D**) (Zhang et al., 2023). Co-occurrence network analysis revealed that rhizosphere fungal networks exhibited higher connectivity, reflecting greater stability, functionality, and adaptability (**Figure 2G**). The rhizosphere, with its abundant resources, supports extensive fungal cooperation and complex networks, whereas the more isolated environment of the root endosphere reduces diversity and limits innately specific taxa to thrive (Wu et al., 2024a).

The results indicated that *Asc*ractions, thus allowing on*omycota* were dominant in the root-associated fungal communities of pear trees across all regions (**Figure 3A**). The prevalence of *Ascomycota* in most plants can be attributed to their strong spore production and rapid growth, which enable them to quickly establish dominance under favorable conditions. Moreover, *Ascomycota* are saprophytes that decomposed recalcitrant organic matter, playing a crucial role in nutrient cycling in the rhizosphere and maximizing nutrient recovery for pear trees *in situ*. In the rhizosphere of YT, many *Leotiomycetes* were present (**Figure 3B**), exhibiting rich species diversity. Some of these groups could produce secondary metabolites with complex structures and broad activities, which have significant potential for development in plant pest control and other applications (Fujita et al., 2021). Furthermore, fungal species identification revealed that 0.8–3.92% of the fungal taxa in the rhizosphere and 0.12–0.83% in the root endosphere were unidentified across the five regions, underscoring a substantial reservoir of undiscovered fungal taxa.

In the rhizosphere of pear trees, undefined and unknown saprotrophic fungi predominated, constituting a significant proportion of the fungal community (**Supplementary Figure S5D**). These fungi contribute to soil nutrient enhancement and fertility by decomposing organic matter. Similarly, in the root endosphere fungal communities across the five regions, saprotrophs and pathotrophs were also dominant (**Supplementary Figure S5E**). This may be due to improper agricultural practices or the fact that pear trees, aged approximately 30 years, have gradually entered a stage of decline, leading to weakened resistance in the root systems of these trees to pathogenic fungi. Under favorable environmental conditions, these pathogens may proliferate rapidly, potentially inducing diseases in pear trees (Hardoim et al., 2015). However, numerous probiotic fungi, such as Ericoid, Mycorrhizal, and Ectomycorrhizal fungi (**Supplementary Figure S5D**), also inhabit the rhizosphere, indicating that this ecosystem retains a certain degree of adaptability and self-regulation (Ward et al., 2021).

### 4.2 Stochastic processes govern the assembly of rhizospheric and root endospheric fungal microbiota at the full-fruiting stage of pear

The assembly of plant fungal communities is crucial for plant adaptability and ecological functions (Xu et al., 2023). βNTI analysis revealed that the composition of fungal communities in the rhizosphere and root endosphere of pear trees was predominantly shaped by stochastic processes (**Figures 4A–C**), aligning with previous studies on the mechanisms underlying the construction of rhizosphere fungal communities in *Phoebe bournei* plantations (Yan et al., 2024). The abundance of nutrients can enhance ecological randomness and reduce competitive pressures, leading to stochastic dominance in fungal assembly under anthropogenic disturbances (Dini-Andreote et al., 2015). Additionally, the stochastic processes governing fungal community assembly varied across different regions of the pear roots. Although fungi produce numerous spores, their limited dispersal constrains the spread of the rhizosphere community, making its assembly more influenced by dispersal limitations (Li et al., 2021). In contrast, the root endosphere, with its smaller and less diverse community, was more susceptible to ecological drift, resulting in greater stochasticity (Fitzpatrick et al., 2020). The assembly process of the fungal community is significantly influenced by the habitat type (*R²* = 0.08, P < 0.001), and the response of the rhizosphere fungal community to the habitat type is stronger than that of the endosphere (Rhizo: *R²* = 0.20, P < 0.001; Endo: *R²* = 0.11, P < 0.001) (**Table 1**). For example, the assembly processes of the rhizosphere fungal communities in YT and QY exhibit remarkable similarities (**Figure 4D**). It is speculated that it may be attributed to the fact that the terrain significantly affects the structure of the rhizosphere fungal community through driving rhizosphere sedimentation, and then regulates its ecological functions. Specifically, the differences in hydrothermal conditions caused by different altitude gradients may prompt changes in the secretion patterns of plant roots, leading to the differentiation of carbon metabolic functions and the ecological filtering effect of allelochemicals in root exudates. Eventually, a spatially heterogeneous community structure is formed, thus promoting the adaptive evolution of plants and the stability of ecosystem functions (Yang et al., 2023). Studies have shown that plant roots can recruit rhizosphere microorganisms in a targeted manner by secreting specific metabolites (Bi et al., 2021), and these microorganisms promote nutrient cycling in the root zone through nutritional interactions (Zhao et al., 2022). In mountainous ecosystems, microbial diversity has been confirmed to maintain the stability of soil organic carbon decomposition (Xu et al., 2021). The YT area, situated in the Yanshan Mountains, may experience geographical isolation and hinder the free migration of species, resulting in a relatively limited species pool. Consequently, during root endosphere community assembly, this limitation allows species with similar characteristics to dominate, leading to a significant contribution of homogeneous selection, second only to ecological drift (Fitzpatrick et al., 2020) (**Figure 4E**).

### 4.3 Effects of rhizosphere soil factors on the assembly of fungal communities in pear trees within different habitats

An increasing number of studies have indicated that various soil characteristics, including soil pH (Naz et al., 2022), soil texture (Girvan et al., 2003), and soil nitrogen availability (Frey et al., 2004), may be related to the changes in the composition of fungal communities (Qiu et al., 2022). Similarly, correlation analysis revealed that the physicochemical properties of the soil were the primary factors driving the assembly of rhizosphere and root endosphere fungal communities in pear trees across regions (**Figure 6**). YT exhibited the highest rhizosphere fungal richness (**Figure 2B**), likely due to its abundant organic matter, which promoted fungal growth. In contrast, BT showed the lowest rhizosphere fungal abundance (**Figure 2B**), possibly due to high soil pH and EC, which may stress fungi and limit their growth (Zhang et al., 2016). Co-occurrence networks analysis of rhizosphere soil fungi demonstrated high connectivity and notable regional differences (**Figure 2G**). For example, rhizosphere fungal network in XJ displayed increased competition, potentially attributed to a high C/N ratio that favors nitrogen-efficient fungi (Yang et al., 2017). The study also revealed significant regional differences in the Sobs and Shannon indices of root endosphere fungi. QY exhibited the highest abundance of root endosphere fungi (**Figure 2C**), likely due to its high AP, high C/N ratio, and weakly acidic environment, which were favorable for saprophytic fungi (Yang et al., 2017). BT showed the highest Shannon index for both rhizosphere and root endosphere fungi (**Figures 2B, C**), potentially reflecting the presence of diverse fungal communities that were adapted to high soil pH stress. Co-occurrence network analysis of root endosphere fungi across regions confirmed high connectivity among species and highlighted regional differences in community structures (**Supplementary Figures S2A, B**). The network structure of endophytic fungi in WX was relatively simple, which may be due to the traditional agricultural management practices in WX, which often involve the extensive use of chemical fertilizers and pesticides. These practices may reduce the diversity of endophytic fungi, leading to a more simplified network structure (Wu et al., 2024b; Ye et al., 2021). Venn diagram analysis revealed that more unique genera were found in the rhizosphere and root endosphere of pear trees in the YT and QY regions, respectively (**Supplementary Figures S4B, C**). This may be due to the variable terrain, altitude, and complex soil types that provide diverse habitats for fungi (Siles and Margesin, 2016). Overall, these findings confirmed that fungal community composition varied significantly across regions, and that the effect of topography on fungal community assembly was less pronounced than the effects of regional types and root compartment niches.

## 5 Conclusions

Stochastic processes dominate the assembly of both rhizosphere and root endosphere fungal communities at the soil-root interface during the full fruiting stage of perennial fruit trees. The composition of fungal communities varies significantly among different regions. In both the rhizosphere and root endosphere fungal communities, the number of genera specific to mountainous regions was larger than those in plain areas and saline-alkali areas. The assembly of root-associated fungal communities in *P. betulifolia* is not only driven by soil physicochemical properties but also influenced by root compartment niche and topography. Moreover, the impact intensity of the root compartment niche is greater than topography. Specifically, the assembly of rhizosphere fungal communities primarily driven by AN and ALP, while the assembly of root endophytic fungi was mainly influenced by pH and SUC. The barrier between the rhizosphere-root endosphere in pear trees exerted a strong filtering effect on the recruitment of specific microorganisms. Our study not only describes the diversity of fungal microbiota in the rhizosphere and endosphere of perennial woody fruit trees, but also elucidates the assembly mechanisms of these microbiota and clarifies the impacts of rhizosphere soil factors on fungal community assembly. These findings provide fundamental data for designing beneficial root-associated microbiomes to enhance fruit tree performance.

## Data Availability

The datasets presented in this study can be found in online repositories. The names of the repository/repositories and accession number(s) can be found below: https://www.ncbi.nlm.nih.gov/, PRJNA1161009, https://www.ncbi.nlm.nih.gov/, PRJNA1160469.

## Glossary

WX: Weixian
XJ: Xinji
BT: Botou
QY: Quyang
YT: Yutian
WCS: Rhizosphere fungi in WX
XCS: Rhizosphere fungi in XJ
BCS: Rhizosphere fungi in BT
QCS: Rhizosphere fungi in QY
YCS: Rhizosphere fungi in YT
WCR: Root endosphere fungi in WX
XCR: Root endosphere fungi in XJ
BCR: Root endosphere fungi in BT
QCR: Root endosphere fungi in QY
YCR: Root endosphere fungi in YT
SM: Soil moisture
OC: Organic carbon
TN: Total nitrogen
TP: Total phosphorus
AN: Alkaline nitrogen
AP: Available phosphorus
AK: Available potassium
EC: Electrical conductivity
C/N: Soil organic carbon/Total nitrogen
ALP: Alkaline phosphatase
UR: Urease
SUC: Sucrase
MAT: Mean annual temperature
MAP: Mean annual precipitation
OTUs: Operational Taxonomic Units
PCoA: Principal Coordinates Analysis
pNPP: p-nitrophenyl phosphate
PCoA: Principal Coordinates Analysis
PERMANOVA: Permutational multivariate analysis of variance
VPA: Variance partitioning analysis
