# Supplementary material for: Specific soil factors drive the differed stochastic assembly of rhizosphere and root endosphere fungal communities in pear trees across different habitats
Source: Front Plant Sci. 2025 Apr 1;16:1549173. doi: 10.3389/fpls.2025.1549173 (PMC11996911; doi:10.3389/fpls.2025.1549173)
Supplement: Supplementary file 1 [file DataSheet1.docx]

Supplementary Material

**Supplementary Figures and Tables**

1. **Supplementary Figures**


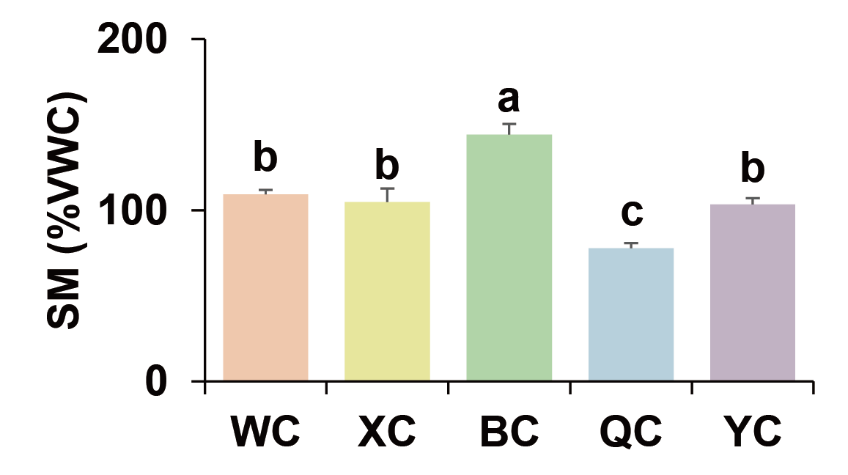


**Supplementary Figure 1.** Differences in soil moisture (SM) of the pear trees rhizospheric soil among different regions. The error bars illustrate the standard error of the mean. Different lowercase letter above the error bars indicates significant difference at *P* < 0.05.


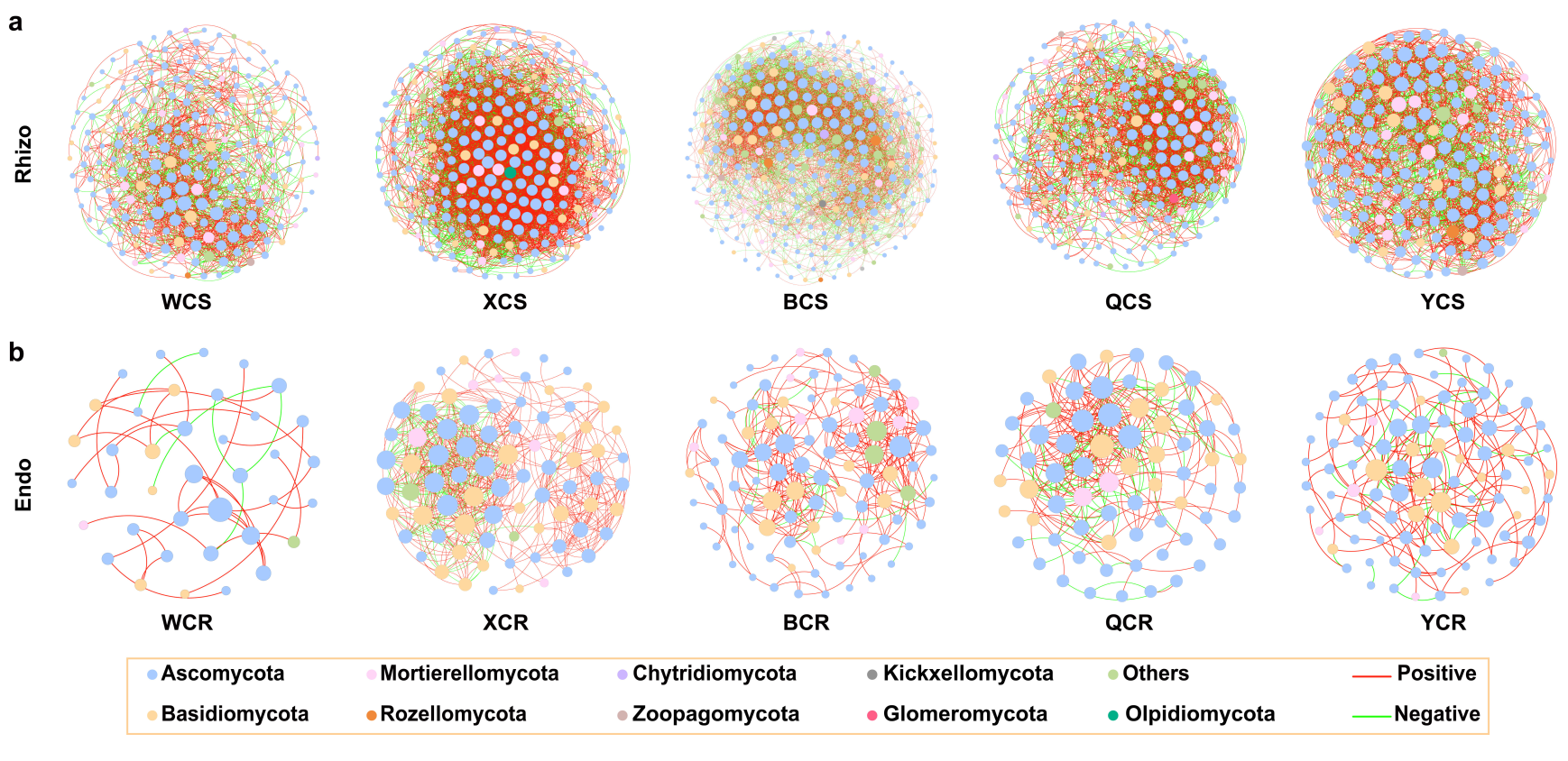


**Supplementary Figure 2.** Co-occurrence networks of fungal communities in the rhizosphere (Rhizo) and root endosphere (Endo). (A) Co-occurrence networks of rhizosphere fungal communities in different regions. (B) Co-occurrence networks of root endosphere fungal communities in different regions. Nodes represent fungal OTUs. Edges represent significant interactive correlations between pairs of OTUs. Node colors represent fungal OTUs species information (phylum level) and the size of nodes corresponds to the relative abundances of specific fungus. Red edges indicate positive relationships, and green edges indicate negative relationships.


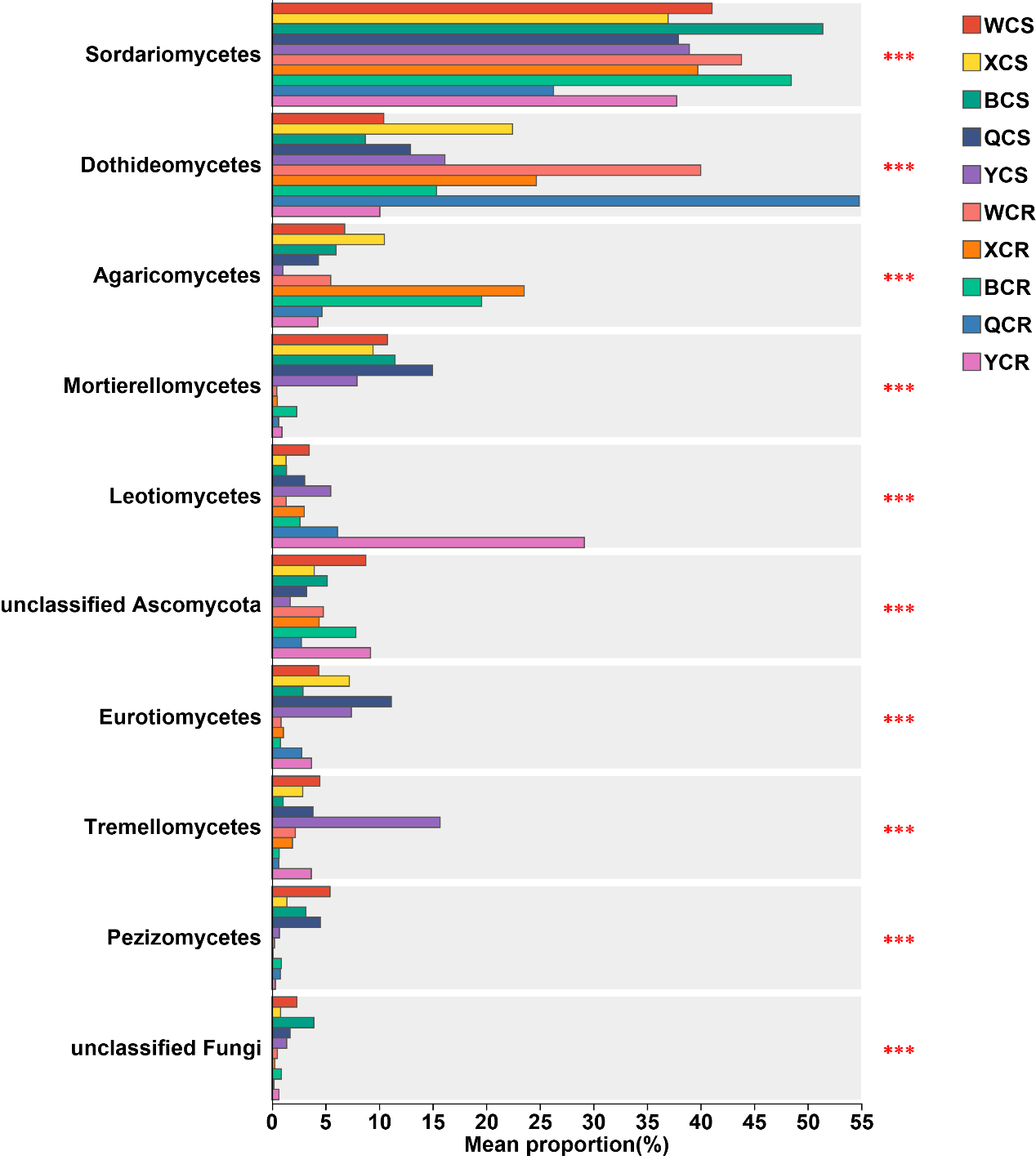


**Supplementary Figure 3.** Taxa differences of fungi between the rhizosphere (S) and root endosphere (R) at the class level across five regions. The vertical axis shows taxa at class level with mean sums in the top 10, and different colored boxes indicate different groups. The horizontal axis represents the average relative abundance of taxa. **P <* 0.05; ***P <* 0.01; ****P <* 0.001, WCS (WCR), XCS (XCR), BCS (BCR), QCS (QCR) and YCS (YCR) represent rhizosphere (root endosphere) fungi in Weixian, Xinji, Botou, Quyang, and Yutian regions, respectively**.**


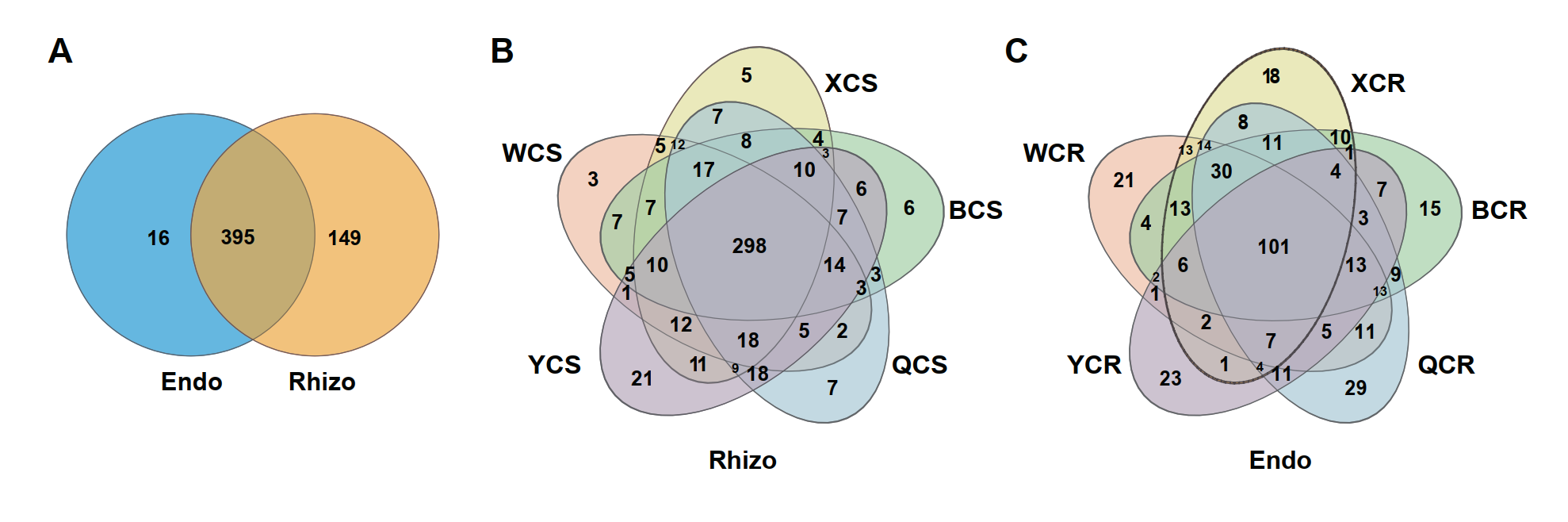


**Supplementary Figure 4.** Venn diagram of the root-associated fungal composition at the genus level. (A) Venn diagram displaying the fungal composition of the rhizosphere (Rhizo) and root endosphere (Endo) at the genus level. (B) Venn diagram displaying the rhizosphere fungal composition in different regions at the genus level. (C) Venn diagram displaying the root endosphere fungal composition in different regions at the genus level.


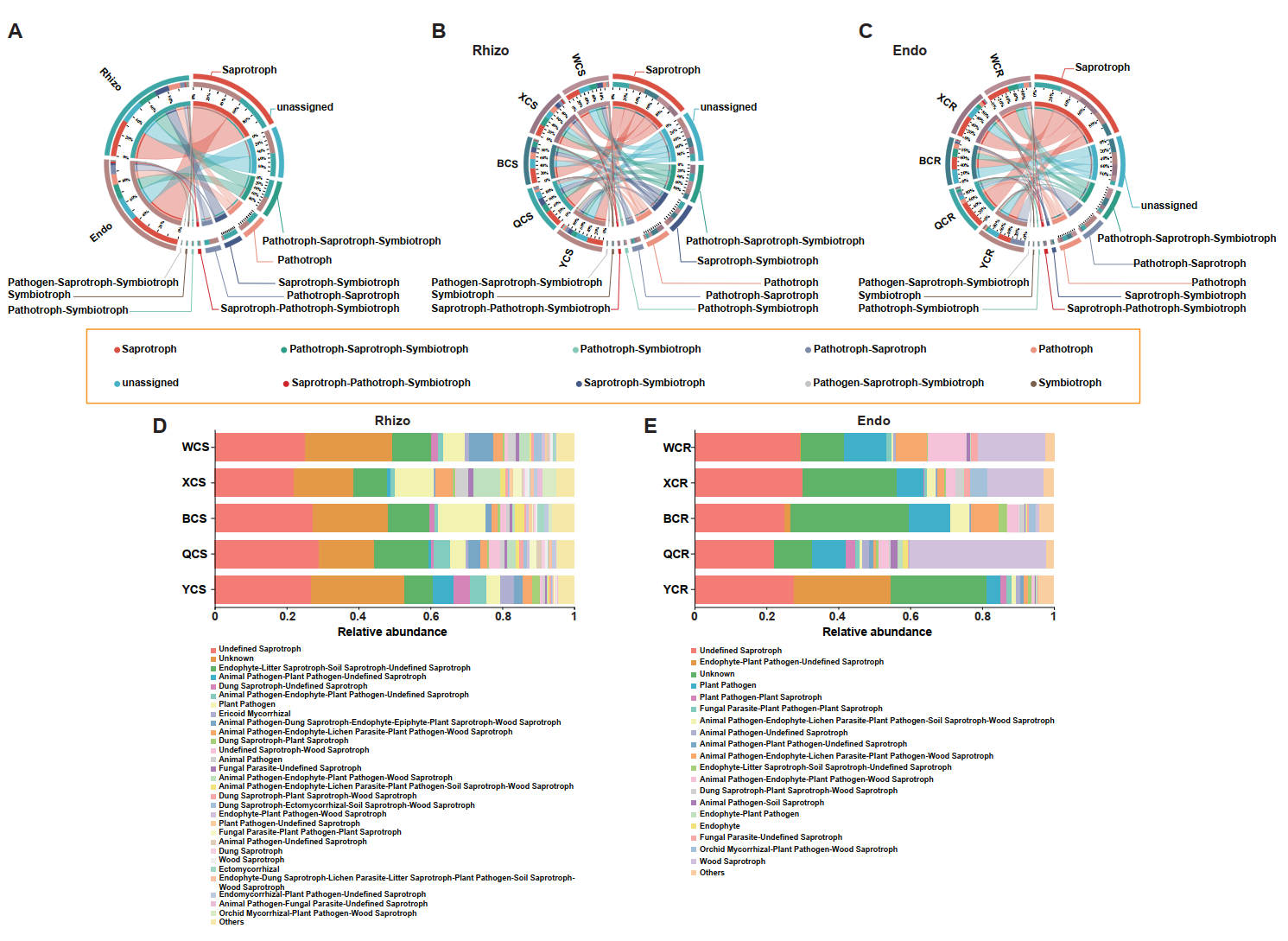


**Supplementary Figure 5.** Functional compositions of the fungal communities inhabiting the rhizosphere (Rhizo) and root endosphere (Endo) fungi. (A) Relative abundance of fungal trophic modes between the rhizosphere and endosphere. The left half circle represents the functional composition of the fungi inhabiting each rhizocompartment. The outer ribbon represents distinct rhizocompartment, the color of the inner ribbon represents different trophic mode, and the length represents the relative abundance of each trophic mode within each rhizocompartment. The right half circle represents the distribution proportion of a certain trophic mode in both rhizocompartments. The outer ribbon represents the trophic modes. The color of the inner ribbon represents the distinct rhizocompartment, and the length represents the overall distribution proportion of a certain trophic mode in both rhizocompartments. One end of the colored strip inside the circle connects to the rhizocompartment (left semicircle), and the other end connects to the trophic mode (right semicircle). Relative abundance of fungal trophic modes in the rhizosphere (B) and root endosphere (C) of different regions. The variation in the relative abundance of rhizosphere (D) and root endosphere (E) fungal functional groups inferred by FUNGuild among different regions.


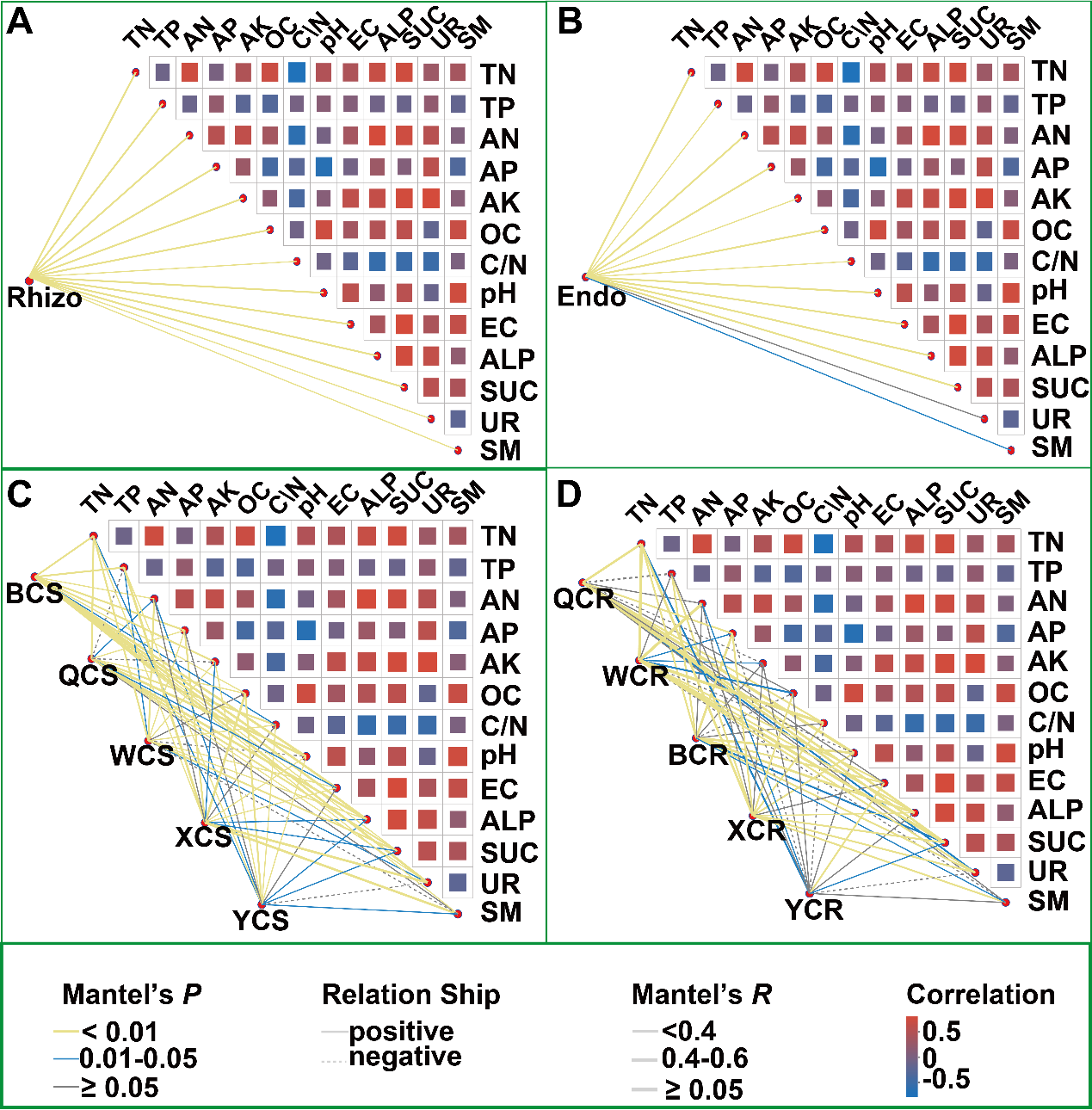


**Supplementary Figure 6.** Association of rhizosphere (Rhizo) and root endosphere (Endo) fungal taxonomic with soil physicochemical properties was analyzed by Mantel tests. (A) Association of rhizosphere fungal taxonomic with soil physicochemical properties was analyzed by Mantel tests. (B) Association of root endosphere fungal taxonomic with soil physicochemical properties was analyzed by Mantel tests. (C) Association of rhizosphere fungal taxonomic in different regions with soil physicochemical properties was analyzed by Mantel tests. (D) Association of root endosphere fungal taxonomic in different regions with soil physicochemical properties was analyzed by Mantel tests. The edge width corresponds to the R value and edge color denotes the statistical significance. The color gradient indicates Pearson correlation coefficients between soil chemical properties.


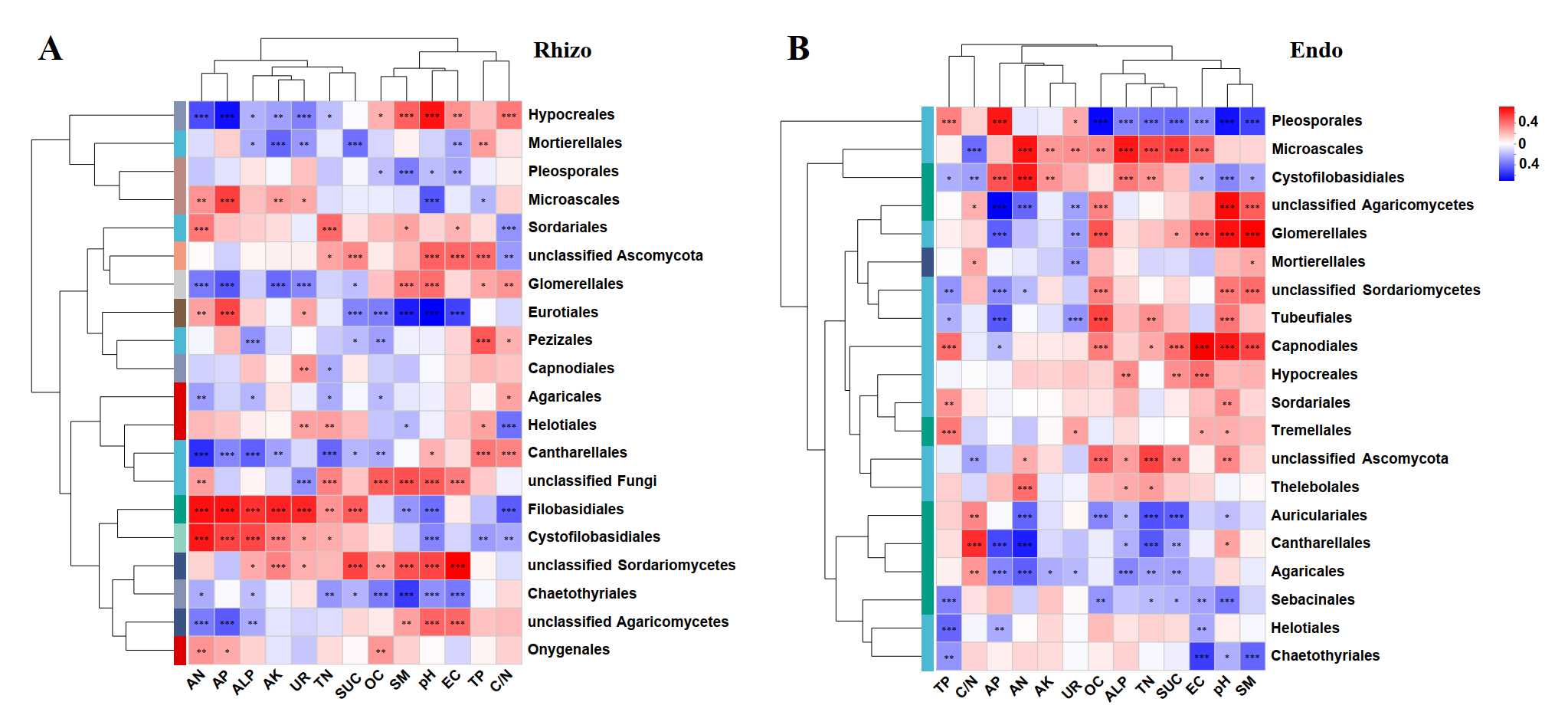


**Supplementary Figure 7.** Spearman’s rank correlation between soil physicochemical properties and relative abundance of major fungal orders in rhizosphere (Rhizo) (A) and root endosphere (Endo) (B) of pear trees. The graphs show the top 20 orders in the relative abundances. * *P* < 0.05, ** *P* < 0.01, and *** *P* < 0.001. The legend on the right is a color range of different R values. Red and blue color indicate positive and negative R values, respectively.

1. **Supplementary Tables**

**Supplementary Table 1** The sampling sites for pear root and rhizosphere soil samples from five regions in Hebei Province. Note. MAT: Mean annual temperature; MAP: mean annual precipitation.

| Region | Longitude | Latitude | MAT (℃) | MAP (mm) | Altitude (m) | Soil pH |
| --- | --- | --- | --- | --- | --- | --- |
| WX | 114°88' E | 36°35' N | 15.0 | 437.6 | 38.9 | 7.4 |
| XJ | 115°31' E | 37°96' N | 15.5 | 443.9 | 14.6 | 7.4 |
| BT | 116°52' E | 38°09' N | 15.0 | 361.8 | 4.4 | 8.2 |
| QY | 114°82' E | 38°57' N | 13.5 | 816.7 | 67.6 | 6.8 |
| YT | 117°59' E | 39°98' N | 12.7 | 1355.8 | 27.4 | 7.4 |

Note. MAT: Mean annual temperature; MAP: mean annual precipitation.

**Supplementary Table 2** Structural attributes of the rhizosphere (Rhizo) and root endosphere (Endo) fungal networks.

| Network | Total | |  | Rhizo | | | | |  | Endo | | | | |
| --- | --- | --- | --- | --- | --- | --- | --- | --- | --- | --- | --- | --- | --- | --- |
|  | Rhizo | Endo |  | WCS | XCS | BCS | QCS | YCS |  | WCR | XCR | BCR | QCR | YCR |
| nodes | 351 | 91 |  | 225 | 247 | 279 | 225 | 175 |  | 38 | 80 | 94 | 69 | 97 |
| edges | 1171 | 123 |  | 1401 | 4434 | 3783 | 2081 | 1933 |  | 38 | 548 | 320 | 267 | 174 |
| Positive links (%) | 87.36 | 99.19 |  | 63.03 | 54.83 | 55.46 | 55.98 | 61.98 |  | 84.21 | 74.27 | 93.12 | 71.91 | 81.03 |
| Negative links (%) | 12.64 | 0.81 |  | 36.97 | 45.17 | 44.54 | 44.02 | 38.02 |  | 15.79 | 25.73 | 6.88 | 28.09 | 18.97 |
| Network density | 0.019 | 0.03 |  | 0.056 | 0.146 | 0.098 | 0.083 | 0.127 |  | 0.054 | 0.173 | 0.073 | 0.114 | 0.037 |
| modularity | 0.7 | 0.828 |  | 0.414 | 0.228 | 0.366 | 0.358 | 0.424 |  | 0.798 | 0.428 | 0.557 | 0.351 | 0.591 |
| Clustering coefficient | 0.497 | 0.517 |  | 0.399 | 0.575 | 0.513 | 0.471 | 0.474 |  | 0.568 | 0.663 | 0.593 | 0.505 | 0.27 |
| Characteristic path length | 4.395 | 3.855 |  | 3.099 | 2.444 | 2.615 | 2.79 | 2.278 |  | 2.011 | 2.661 | 3.347 | 3.33 | 4.16 |
